# Administration of *Mycobacterium leprae* rHsp65 Aggravates Experimental Autoimmune Uveitis in Mice

**DOI:** 10.1371/journal.pone.0007912

**Published:** 2009-11-19

**Authors:** Eliana B. Marengo, Alessandra Gonçalves Commodaro, Jean Pierre S. Peron, Luciana V. de Moraes, Fernanda C. V. Portaro, Rubens Belfort, Luiz Vicente Rizzo, Osvaldo Augusto Sant'Anna

**Affiliations:** 1 Department of Immunology, Institute of Biomedical Sciences, University of São Paulo, São Paulo, Brazil; 2 Vision Institute, Federal University of São Paulo, São Paulo, Brazil; 3 Immunochemistry Laboratory, Instituto Butantan, São Paulo, Brazil; 4 Albert Einstein Jewish Institute for Education and Research, São Paulo, Brazil; Tel Aviv University, Israel

## Abstract

The 60kDa heat shock protein family, Hsp60, constitutes an abundant and highly conserved class of molecules that are highly expressed in chronic-inflammatory and autoimmune processes. Experimental autoimmune uveitis [EAU] is a T cell mediated intraocular inflammatory disease that resembles human uveitis. Mycobacterial and homologous Hsp60 peptides induces uveitis in rats, however their participation in aggravating the disease is poorly known. We here evaluate the effects of the *Mycobacterium leprae* Hsp65 in the development/progression of EAU and the autoimmune response against the eye through the induction of the endogenous disequilibrium by enhancing the entropy of the immunobiological system with the addition of homologous Hsp. B10.RIII mice were immunized subcutaneously with interphotoreceptor retinoid-binding protein [IRBP], followed by intraperitoneally inoculation of *M. leprae* recombinant Hsp65 [rHsp65]. We evaluated the proliferative response, cytokine production and the percentage of CD4^+^IL-17^+^, CD4^+^IFN-γ^+^ and CD4^+^Foxp3^+^ cells *ex vivo*, by flow cytometry. Disease severity was determined by eye histological examination and serum levels of anti-IRBP and anti-Hsp60/65 measured by ELISA. EAU scores increased in the Hsp65 group and were associated with an expansion of CD4^+^IFN-γ^+^ and CD4^+^IL-17^+^ T cells, corroborating with higher levels of IFN-γ. Our data indicate that rHsp65 is one of the managers with a significant impact over the immune response during autoimmunity, skewing it to a pathogenic state, promoting both Th1 and Th17 commitment. It seems comprehensible that the specificity and primary function of Hsp60 molecules can be considered as a potential pathogenic factor acting as a whistleblower announcing chronic-inflammatory diseases progression.

## Introduction

Uveitis is an inflammation of the uvea, a layer of the eye located between the sclera and the retina. Uvea includes the iris, ciliary body, and choroid [1]. The animal model for human uveitis has experimental autoimmune uveitis [EAU] that also resembles that seen in diseases such as sympathetic ophthalmia, birdshot retinochoroidopathy, Vogt-Koyanagi-Harada's disease, and Behcet's disease [BD] [2]. EAU is an organ-specific T-cell-mediated disease that can be induced in susceptible animals such as non-human primates and rodents. Induction is done with immunization using retinal antigens like interphotoreceptor retinoid-binding protein [IRBP] or with S-antigen [arrestin]. It can also be done by adoptive transfer of IRBP or arrestin-specific T-cells [2], [3], [4]. The disease is characterized by vasculitis and granuloma formation in the neural retina, destruction of photoreceptor cells, and blindness [2], [4].

The 60kDa heat shock protein family, HSP60, constitutes an abundant and highly conserved class of molecules. They prevent misfolding and promote the refolding and proper assembly of unfolded polypeptides generated under physiologic and stress conditions. The *Mycobacterium leprae* Hsp65 [*M. leprae* Hsp65] is part of the HSP60 family denominated chaperonins. It is considered as one of the major immuno-reactive proteins in mycobacteria [5]. Mycobacterial Hsp65 shares approximately 50% amino acid identity with its mammalian homologue. The Hsp60 family comprises molecules that are immunodominant in several infectious processes [6], and which participate in pro-inflammatory events associated to pathologies such as arthritis, atherosclerosis, type I diabetes, and autoimmune demyelination [7], [8], [9]. As for its association with eye disease more specifically, higher anti-Hsp65 antibody titers were correlated to the retinopathy seen in type I diabetes patients [10].

Mycobacterial and homologous Hsp60 peptides can induce experimental uveitis in rats [11], [12] and Hsp60 and anti-Hsp60 may be involved in the pathogenesis of the ocular manifestations of Behcet's disease and acute anterior uveitis [13], [14]. However, their participation in aggravating the disease is poorly known.

Although the etiology of autoimmune diseases still remains unclear, it is believed that molecular mimicry is a contributing factor [15]. The presence of antibodies and T cells that are reactive to Hsp60 or Hsp65 in pathophysiological conditions suggests that these proteins play an important role as cellular targets in autoimmunity due to an existing similarity between the bacterial antigen and the autologous protein [16]. On the other hand, high anti-Hsp60/65 antibody titers were not found to be restricted to disease conditions and could also be detected in supposedly healthy individuals as they aged [17], [18]. Indeed, autoantibodies can be indicators of susceptibility to the future development of various autoimmune diseases [19], [20], [21], [22].

We previously showed that passive administration of wild type *M. leprae* Hsp65 interfered with endogenous equilibrium by enhancing the entropy of the immunobiological system, as expressed by the early death of the systemic lupus erythematosus [NZBxNZW]F_1_ experimental mice [23]. We here applied a similar approach of inducing disequilibrium of physiological and immune states by adding homologous Hsp to evaluate the effects of the *M. leprae* Hsp65 in the development/progression of EAU by evaluating the autoimmune response against the eye. For that we analyzed the proliferative response, cytokine and antibody production in the B10.RIII experimental mice submitted to immunization and administration of Hsp65. The findings were compared to the histopathological analysis of the eye.

## Results

### Administration of *M. leprae* rHsp65 Increased EAU Scores

**Figure 1:**
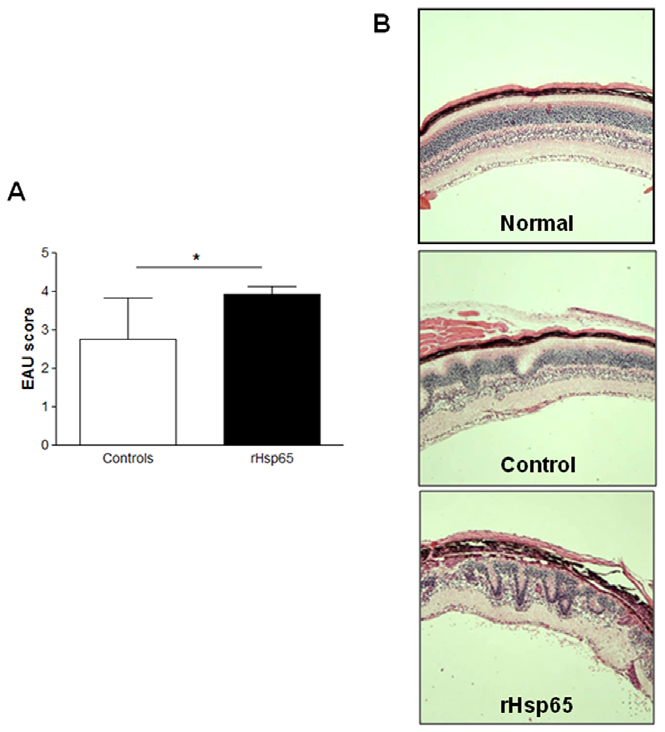
Administration of *M. leprae* rHsp65 increased EAU scores. [*A*]: B10.RIII mice were immunized with 161–180 IRBP peptide followed by injection with rHsp65 [i.p.]. Eyes were collected for histopathology 21 days later. EAU scores were assigned on a 0 to 4 scale. [*B*]: Histopathological findings: a normal retinal architecture corresponding to non immunized *naïve* mice [*normal*]; IRBP immunized mice [average score 3; *control*]; mice immunized with IRBP followed by rHsp65 inoculation [average score 4; *rHsp65*]. Representative photographs [hematoxilin-eosin staining] of three independent experiments with n = 6–8 mice/group. Magnification: 200x.

To evaluate the influence of rHsp65 in the progression of EAU, B10.RIII mice were induced to develop uveitis by immunization with IRBP followed by inoculation with rHsp65, which occurred in the same day. Control mice showed moderate to severe signs of EAU according to histopathologic examination done on day 21. These signs were retinal disorganization, presence of inflammatory cells in the vitreous, vasculitis and granuloma formation [Figures 1A and 1B, *control group*]. Conversely, rHsp65-inoculated animals presented a more pronounced inflammatory response, with total disorganization of the retinal layers associated to detachment of the retina. Intense retinal folds, inflammatory infiltrating cells in the vitreous, vasculitis and granuloma formation were also observed [Figures 1A and 1B, *rHsp65 group*]. B10.RIII mice immunized with IRBP emulsified in incomplete Freund adjuvant plus PTX or immunized in CFA without PTX, even receiving rHsp65, do not express EAU [data not shown]. These indicate that Hsp have any commitment in the initiation process of this organ-specific autoimmune disease.

### Analysis of Cytokine Levels

**Figure 2:**
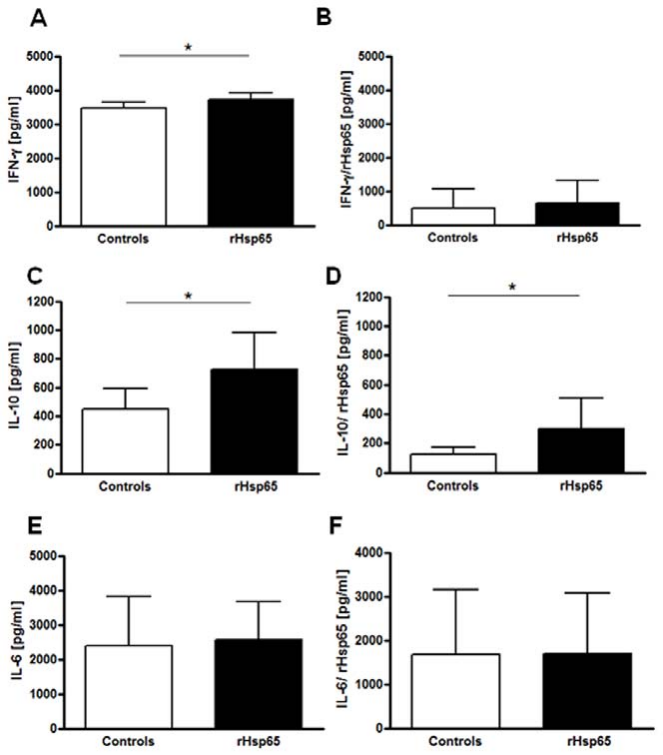
rHsp65 inoculation increased IFN-γ and IL-10 levels. Draining lymph nodes cells from either rHsp65 inoculated or control mice were harvested at day 21 and stimulated *in vitro* [10^6^ cells/ml] with 30 µg/ml IRBP [*A, C, E*] and 20 µg/ml *M. leprae* rHsp65 [*B, D, F*]. After 48 hours, IFN-γ [*A, B*], IL-10 [*C, D*] and IL-6 [*E, F*] levels were determined by ELISA. Data represent three independent experiments with n = 5 mice/group [*p<0.05 versus control group; *t*-test analysis].

**Figure 3:**
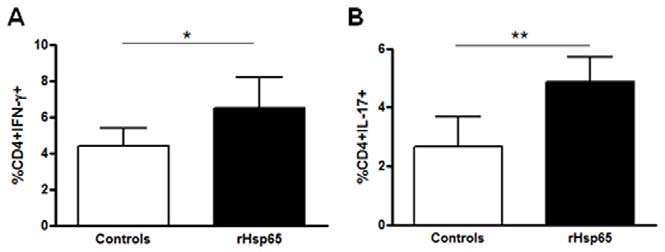
Expansion of CD4^+^IFN-γ^+^ and CD4^+^IL-17^+^ T cells after rHsp65 inoculation. Cells from draining lymph nodes collected at day 21 after immunization were labeled with anti-CD4 mAb, fixed and permeabilized, stained intracellularly and analyzed by flow-cytometry evaluating the CD4, IFN-γ and IL-17 expression. Results are expressed in mean ± SD. Data represent three independent experiments with n = 5 mice/group [*p<0.05; **p<0.01 versus control group; *t*-test analysis].

Draining lymph node cells from either control or rHsp65-inoculated mice were harvested on day 21 after immunization and assayed for cytokine secretion under IRBP and rHsp65 stimulating conditions. We observed an increased IFN-γ secretion by IRBP re-stimulated cells, which was associated with a higher expansion of CD4^+^IFN-γ^+^ T cells [Figures 2A and 3A, *p*<0.05] in the rHsp65 group. These findings corroborate with our clinical findings, in which an increase in the EAU score was observed. On the other hand, no changes were observed in IFN-γ after re-stimulation with rHsp65 [Figure 2B].

Surprisingly, rHsp65 administration resulted in an increased IL-10 production after stimulation with IRBP and with rHsp65 [Figures 2C and 2D, *p*<0.05]. No alterations in IL-6 production levels were observed after IRBP and rHsp65 stimulation [Figures 2E and 2F].

### rHsp65 Expanded IL-17 and IFN-γ CD4^+^T Cell Population

**Figure 4:**
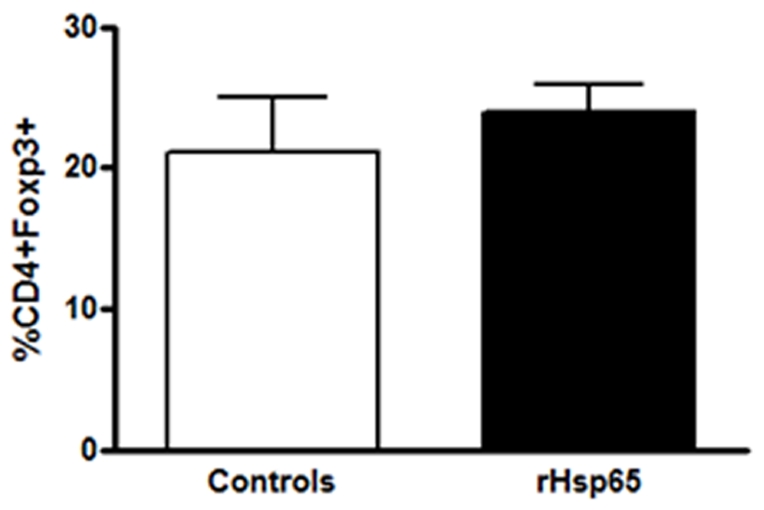
rHsp65 showed no effect on CD4+Foxp3+ T cells. Cells from draining lymph nodes were harvested at day 21 after immunization, stained and analyzed by flow-cytometry evaluating the CD4 and Foxp3 expressions. Results are expressed in mean ± SD. Data represent three independent experiments with n = 5 mice/group.

Because the Th17 cells recently described are believed to play a relevant role in the pathogenesis of autoimmunity [24], [25], [26] and the expression of Th1 is a characteristic phenotype of EAU, we evaluated the percentage of both Th1 and Th17 cells from lymph nodes of EAU mice inoculated with rHsp65 and compared to controls. Intracellular staining for IFN-γ and IL-17 revealed that rHsp65 injection induced expansion of both CD4^+^ T cell populations [Figure 3]. Concerning T cell subsets, it is possible that the higher disease scores observed in the rHsp65 group would lead to an impaired percentage of T regulatory cells in the periphery. However, this was not the case, as no differences in the percentages of CD4^+^Foxp3^+^ T cells were observed [Figure 4].

### Anti-IRBP IgG2a Levels Decreased after rHsp65 Administration

**Figure 5:**
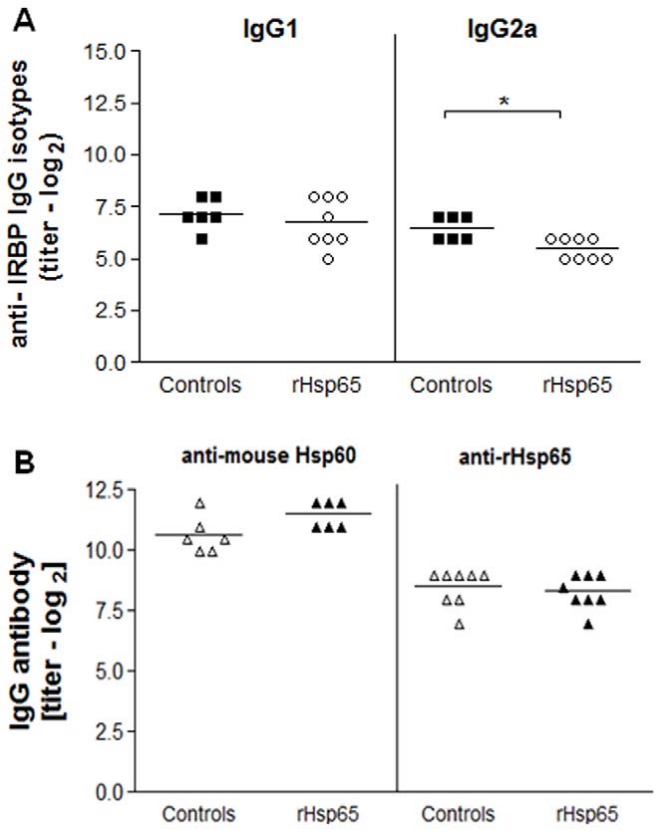
rHsp65 modulated anti-IRBP IgG2a levels. Serum levels of anti-IRBP IgG1 and IgG2a isotypes [*A*] and IgG anti-mouse Hsp60 and anti-rHsp65 [*B*] antibody production in IRBP-immunized mice either or not inoculated with rHsp65. Antibody levels were determined by ELISA at day 21 after immunization. Results are expressed in titers ± SD in each experimental group. Data represent three independent experiments with n = 6–8 mice/group [*p<0.05 versus control group; *t*-test analysis].

We examined the effects of rHsp65 inoculation on IRBP and Hsp60/65 specific IgG antibody production. As shown in Figure 5A, anti-IRBP IgG2a levels decreased in the sera of the rHsp65 mice group when compared to control [*p*<0.05]. No significant differences in the levels of anti-IRBP IgG1 antibodies were observed.

Production of anti-mouse Hsp60 and anti-Hsp65 antibody showed no difference between test and control in the mice sera [Figure 5B]. However, production of anti-Hsp60 antibody [both in the test group and in controls] presented titers that were approximately 5.6-fold higher than those of anti-Hsp65 antibodies.

## Discussion

Hsp60 are highly conserved molecules inducible by all forms of cellular stress. In spite of the best known function they have as chaperonin, Hsp are also important players in the control of the immune response, being target by Hsp-specific T cells and antibodies in healthy subjects and also in chronic-inflammatory processes including autoimmune diseases [6], [27], [28], [29]. Hsp have shown a dual role in immune-mediated disorders, being involved in the induction and propagation of autoimmune diseases as well as in suppressing them [30]. The precise mechanisms underlying these divergent Hsp60-mediated responses are not defined. It has been suggested that the increased expression of endogenous Hsp molecules in different stress conditions can propagate the ongoing inflammation and also constitutes an attractive target for T cells and antibodies that are induced by extracellular [self or foreign] Hsp. In addition, inflammation can alter the antigen processing for Hsp and reveal new epitopes which can prime an immune response and lead to pathological status [31]. Furthermore, these molecules activate innate immunity through macrophages and dendritic cells, which in turn trigger the development of adaptive immune response to self Hsp60 by extracellular Hsp60/65. Besides that, anti-Hsp65 antibodies can also be a potent pathological trigger [7], [32]. The immune response to Hsp can be mined for information on the state of the immune system [33], [34]; in altered conditions, Hsp and their antibodies can act as biomarkers during postnatal life [20], [35].

In this study we showed that a single administration of *M. leprae* rHsp65 in the highly susceptible B10.RIII immunized mouse was capable to increase the scores of uveitis when compared to controls [*p*<0.05]. Also, rHsp65-inoculated animals presented a more pronounced inflammatory response, with total disorganization of the retinal layers associated to its detachment. Intense retinal folds, inflammatory infiltrating cells in the vitreous, vasculitis and granuloma formation were also observed.

Most reports that attempted to correlate Hsp60 with uveitis were limited to studies done with systemic Behcet's disease [11], [36], [37], [38]; increased T and B cell activity against Hsp60 and Hsp65 has been documented in these patients [11], [39], [40], [41]. The inoculation of bacterial Hsp65 peptides either subcutaneously or orally, or their human analogues, induced uveitis in Lewis rats by self-reactive T cells specific to these peptides. Although the Hsp60 family members share a common basic antigenicity, they differ in the reactivity to the anti-Hsp60 antibodies present in the sera of BD patients [42]. Cancino-Diaz and collaborators hypothesized that the high levels of anti-bacterial Hsp60 antibodies found in patients with uveitis was produced during episodes of bacterial infection that may have triggered an autoimmune reaction through a mechanism of molecular mimicry between the human and microbial Hsp. This suggests also that an immune cross-reaction between retinal and Hsp molecules and a related autoimmune response may be involved in the development of BD [41].

Many studies showed Hsp60 as a ligand for TLR-2 and TLR-4, suggesting a role for Hsp60 as an immunological and endogenous sign of danger that leads to rapid inflammatory cytokine release and enhancement of adaptive Th1-type responses [43], [44], [45], [46]. Genetic susceptibility to EAU is associated to a dominant Th1-type response to the uveitogenic retinal antigen; the effector T cells possess a Th1-like phenotype that is expressed as high IFN-γ and low IL-4 cytokines production [47], [48]. In our study, the levels of IFN-γ secretion and the percentage of CD4^+^IFN-γ^+^ and CD4^+^IL-17^+^ cells were significantly higher in the rHsp65 group than in control mice. IL-17-producing CD4^+^ T cells or Th17 constitute a newly identified inflammatory cell population, which is critically involved in EAU [24], [25], [49] suggesting that rHsp65 aggravates EAU pathology by expanding antigen-specific Th1 and Th17 cells. Knowing that IL-6 is associated with the production of the Th17 effector phenotype [50] and that the cytokine is responsible, at least partially, for an ocular inflammation in which differentiation of Th17 is IL-6-dependent [26], we expected to see an increased secretion of IL-6 in rHsp65-inoculated mice. Expansion of Th17 did not reflect in a higher production of IL-6 in the rHsp65 group, when compared to controls.

The analysis of the kinetics of cytokine secretion and other time-points may have showed otherwise and explain the lack of differential IL-6 production, in the rHsp65 group and emerge an increase of IL-10 production by IRBP and Hsp65-specific cells. Although IL-10 has been implicated in the progression control of immune-mediated diseases and in the maintenance of tolerance [51], [52], in our study it did not correlate with suppression, since the EAU score was higher in animals that received Hsp65. Concerning T cell subsets, it is possible that the higher disease scores observed in the rHsp65 group would lead to an impaired percentage of T regulatory cells in the periphery. However, this was not the case, as no differences in the percentages of CD4^+^Foxp3^+^ T cells were observed. The elevated IL-10 production in this context may works as an attempt to control the balance Th2/Th1 during the intense inflammatory response caused by the administration of rHsp65, other than being an attempt to regulate the response to self antigens. The higher IL-10 cytokine production and the decrease in IgG2a anti-IRBP levels in rHsp65-inoculated mice reinforce the notion of reacquired homeostasis states.

No significant differences in the production of anti-mouse Hsp60 and anti-Hsp65 antibody levels in both rHsp65 and control groups were observed [Figure 5B]. However, the anti-Hsp65 antibody production was higher in B10.RIII immunized than naïve mice, which showed basal production [data not shown]. The high anti-Hsp60/65 antibody titers were not found to be restricted to disease conditions. That they were detected in supposedly healthy individuals [17], [18] may be due to the fact that the pathophysiologic status of the individuals were not deeply investigated. However, we cannot rule out that anti-Hsp65 antibodies are involved in vascular damage and consequently aggravate ocular disease in Hsp65-inoculated mice, since EAU can present vasculitis and granuloma formation in the neural retina [2], [4]. Indeed, it was reported that the anti-Hsp60 autoantibodies mediates endothelial cell cytotoxicity [53]. The possible correlation between anti-Hsp65 antibodies and vascular lesions and the physiopathological mechanisms involving pro-inflammatory cytokines in the serum of inoculated and control mice may clarify the role of these soluble molecules in the severity of uveitis.

Hsp have been implicated in the induction and propagation of autoimmunity in several diseases. Our findings demonstrate that this may also be true in uveitis and it opens the possibility that the blockage of endogenous produced Hsp, whose expression should be enhanced during autoimmunity, may present an interesting target for immunotherapy of this syndrome of the eye. Furthermore, in infectious diseases of the eye, such as toxoplasmosis, recurrence of lesions often go unexplained and because Hsp are highly conserved in nature it is entirely possible that the pathogen's Hsp may contribute to disease flare up.

Through an original approach based in the Prigogine's principle [54], [55], the pathophysiological role of the rHsp65 of *M. leprae* was also evaluated in the *in vivo* spontaneous Systemic Lupus Erythematosus [SLE] model. It is assumed that the immune system keeps the order state at the cost of irreversibility and that in faster reiterated rounds of stimulation the system is not able to come back to its original state [23]. Both in the organ-specific EAU and in the systemic SLE, these findings strongly support that the over expression of the Hsp family and the altered microenvironmental state in pathologic conditions can modify the antigen processing of Hsps molecules, providing it a toxin function.

The adaptive management of biological systems according to environmental changes is mandatory for the organism survival and the Hsp molecules may intervene in the quantitative and qualitatively immune phenotypes submitted to independent polygenic controls, such as inflammatory and antibody responsiveness or immunological tolerance [56], [57], [58], [59]. With the aggravation of an autoimmune process, the organism is incapable to regenerate the affected tissues, and unable to return to the original immune state. Thus, aggravation of an autoimmune process can be defined as irreversible phenomena [23]. Despite the strategy of balancing the immune response to solve the acute ocular inflammation, the immune system tries to regulate the Th1 proinflammatory response through the production of immunosupressor cytokines. The B10.RIII immunized mice followed by rHsp65 administration were unable to revert to its original visual acuteness, most likely because of a cumulative and irreversible effect of the immune system.

Our data suggest that Hsp65 has pathological significance during the ocular autoimmune disease by increasing the immune response associated with an expansion of CD4^+^IFN-γ^+^ and CD4^+^IL-17^+^ T cells, probably by skewing it to a pathogenic state, promoting both Th1 and Th17 commitment.

These findings can be used for a better understanding of the relationship between self molecules, such as Hsp, and the immune response under physiological and pathological conditions. The specificity and primary function of Hsp60 can be considered as a potential pathogenic factor that also may act as a whistleblower announcing chronic-inflammatory diseases progression. It may be consider the immune system as a broken mirror in which identical or similar images, or their own reflections, distinctly recognized or blurred. In this sense, depending on the pathophysiological conditions, unpredictable immune responsiveness will be evoked by cumulative and temporal effects.

## Materials and Methods

### Antigens and Reagents

Peptide SGIPYIISYLHPGNTILHVD representing residues 161–180 of IRBP was synthesized using the Fmoc procedure [60] in a Shimadzu PSSM8 peptide synthesizer [Shimadzu, Tokyo, Japan]. The Fmoc-amino acids were purchased from Novabiochem [Nottingham, UK]. *Bordetella pertussis* toxin and Complete Freund Adjuvant [CFA] were purchased from Sigma-Aldrich [St. Louis, MO, USA]. Purified mouse Hsp60 [ESP-741] was obtained from Stressgen Bioreagents [San Diego, CA, EUA].

### Expression and Purification of the Recombinant *Mycobaterium leprae* Hsp65 in *Escherichia coli*

Clone pIL161 containing the coding sequence of the *M. leprae* Hsp65 was kindly given by Prof. Célio L. Silva from University of Sao Paulo, Ribeirão Preto, Brazil. The clone was amplified in *E. coli* DH5α cells. Expression of the recombinant wild-type Hsp65 protein [rHsp65] was performed as described in [61]. The rHsp65 present in the bacterial pellet was solubilized with 6 M urea and submitted to preparative SDS-PAGE gel. Elution of the Hsp65 band from the polyacrylamide gel slice was performed to obtain the purified protein [23]. Concentration of the recombinant Hsp65 protein was determined using the Bradford assay [62].

### Animals

Six to eight weeks old B10.RIII mice were obtained from the animal facilities at the University of Sao Paulo, Brazil. All animals were housed under specific pathogen-free conditions and handled under ethical conditions. The Animal Care Committee of the Institute of Biomedical Sciences at the University of Sao Paulo approved all the procedures used in this study.

### Induction of EAU and rHsp65 Treatment

Mice were immunized subcutaneously [s.c.] at the base of the tail with 30 µg of 161–180 IRBP peptide emulsified in 0.2 ml CFA [v/v] and injected intraperitoneally [i.p.] with 0.5 µg of *Bordetella pertussis* toxin [PTX] in 0.1 ml, as additional adjuvant. Following immunization, a group of 6 to 8 animals was inoculated with 2.5 µg of *M. leprae* rHsp65 in 0.2 ml of phosphate buffer saline pH 7.4 [PBS] by i.p. route. At day 21 after immunization, rHsp65 inoculated and control mice were sacrificed. The histopathology of the eye, cellular phenotype, cytokines and anti–IRBP and anti-Hsp60/Hsp65 antibody production were then analyzed.

### Histopathology EAU

Eyes were collected and prepared for histopathological evaluation at day 21 after immunization. The eyes were immersed for 1 h in phosphate-buffered glutaraldehyde 4%, transferred into phosphate-buffered formaldehyde 10% for 24 h, and to ethanol 70% until processing. Fixed and dehydrated tissues were embedded in paraffin wax; 4–6 µm sections were cut through the papillary-optic nerve plane. Sections were stained by hematoxylin and eosin.

Presence of disease was evaluated in a double-blinded fashion by examining six sections cut at different levels in each eye. Severity of EAU was scored on a scale of 0 [no disease] to 4 [maximum disease] in half-point increments, according to a semi quantitative system described previously [2] and which considers the lesion type, size, and number. The minimal EAU score is characterized by inflammatory cell infiltration of the ciliary body, choroids, or retina [EAU score 0.5]. Progressively higher scores were assigned according to the amount, in the tissues, of discrete lesions such as vasculitis, granuloma formation, retinal folding and/or detachment and photoreceptor damage.

### Determination of Cytokine Production

Draining lymph node cells harvested at day 21 after immunization were cultured in 24-well plates [10^6^ cells/ well] and stimulated with 30 µg/ml IRBP or 20 µg/ml *M. leprae* rHsp65. Supernatants were collected for cytokine analysis after 48 h and stored at −80°C until assayed. The IFN-γ, IL-10 and IL-6 cytokines were detected with the ELISA kit from eBiosciences [San Diego, CA, USA] or BD Pharmingen [La Jolla, CA, USA] according to the manufacturer's instructions.

### Flow Cytometry

Lymph node cells were incubated for 20 min on ice with Fc block [hamster anti-mouse CD16/CD32, clone 24G2; BD Pharmingen, San Diego, CA, USA] and labeled with anti-CD4 PE-Cy5 [BD Pharmingen, San Diego, CA, USA], anti-CD25 PE [BD Pharmingen, San Diego, CA, USA] or matched isotype controls for an additional 30 minutes. All antibodies were obtained from BD Pharmingen [San Diego, CA, USA]. For intracellular Foxp3 staining, cells were fixed and permeabilized according to the manufacturer's procedure for the staining set [eBioscience, San Diego, CA, USA] and labeled with PE-conjugated anti-mouse Foxp3 antibody. Cells were washed and then analyzed by flow-cytometry.

For intracellular detection of IFN-γ and IL-17, cells were collected and stimulated for 5 h with 50 ng/ml phorbol myristate acetate [PMA] [Sigma-Aldrich, St. Louis, MO, USA] and 750 ng/ml ionomycin [Calbiochem, La Jolla, CA, USA] in the presence of Golgistop at the recommended concentrations [BD Pharmingen, San Diego, CA, USA]. Another group of cells was not stimulated and used as a control. Both groups of cells [stimulated and control] were stained with fluorescein conjugated anti-CD4+, fixed and permeabilized with Cytofix/Cytoperm solution [BD Pharmingen, San Diego, CA, USA] and then labeled with FITC-conjugated anti-IFN-γ and PE-conjugated anti-IL-17 [BD Pharmingen, San Diego, CA, USA]. Samples were acquired on a FACSCalibur [BD Pharmingen, San Diego, CA, USA] and data were analyzed with CellQuest-Pro software [BD Pharmingen, San Diego, CA, USA].

### Titration of Anti-IRBP, Anti-mouse Hsp60 and Anti-Hsp65 Antibodies

Levels of anti–IRBP, anti-Hsp60 and anti-Hsp65 IgG or IgG1 and IgG2a isotypes were determined in control and in rHsp65 inoculated mice sera, by ELISA. Briefly, 96–well microtiter plates [Nunc MaxiSorp, Roskilde, Denmark] were coated with 1 µg/well of one of the following: IRBP, *Mycobacterium leprae* rHsp65, or mouse Hsp60, diluted in 0.1 M NaHCO_3_^-^ pH 9.6, overnight at 4°C. Plates were washed three times with PBS containing 0.05% Tween 20 [PBS–T]. Further steps were performed as described in [23]. Serum was serially diluted starting at 1∶16. Peroxidase–labeled anti-mouse IgG [1∶2500], IgG1 [1∶1000] or IgG2a [1∶1000] were obtained from BD Pharmingen [San Diego, CA, USA]. The antibody titers were expressed as log_2_ of the reciprocal serum dilution giving an absorbance value of 20% of the saturation level.

### Statistical Analysis

Results are expressed as mean values ± standard deviation. The statistical significance was determined by unpaired t-test and set at *p*<0.05 using the GraphPad Prism 4.0 software [La Jolla, CA, USA].
